# Breaking cover: neural responses to slow and fast camouflage-breaking motion

**DOI:** 10.1098/rspb.2015.1182

**Published:** 2015-08-22

**Authors:** Jiapeng Yin, Hongliang Gong, Xu An, Zheyuan Chen, Yiliang Lu, Ian M. Andolina, Niall McLoughlin, Wei Wang

**Affiliations:** 1 Institute of Neuroscience, State Key Laboratory of Neuroscience and Key Laboratory of Primate Neurobiology, Shanghai Institutes for Biological Sciences, Chinese Academy of Sciences, Shanghai 200031, People's Republic of China; 2 Faculty of Life Science, University of Manchester, Manchester M13 9PT, UK

**Keywords:** camouflaged animal motion, macaque V1, V2, and V4, optical imaging, direction-selective neurons, orientation-selective neurons, spatio-temporal energy model

## Abstract

Primates need to detect and recognize camouflaged animals in natural environments. Camouflage-breaking movements are often the only visual cue available to accomplish this. Specifically, sudden movements are often detected before full recognition of the camouflaged animal is made, suggesting that initial processing of motion precedes the recognition of motion-defined contours or shapes. What are the neuronal mechanisms underlying this initial processing of camouflaged motion in the primate visual brain? We investigated this question using intrinsic-signal optical imaging of macaque V1, V2 and V4, along with computer simulations of the neural population responses. We found that camouflaged motion at low speed was processed as a direction signal by both direction- and orientation-selective neurons, whereas at high-speed camouflaged motion was encoded as a motion-streak signal primarily by orientation-selective neurons. No population responses were found to be invariant to the camouflage contours. These results suggest that the initial processing of camouflaged motion at low and high speeds is encoded as direction and motion-streak signals in primate early visual cortices. These processes are consistent with a spatio-temporal filter mechanism that provides for fast processing of motion signals, prior to full recognition of camouflage-breaking animals.

## 1. Introduction

**Figure 1.**
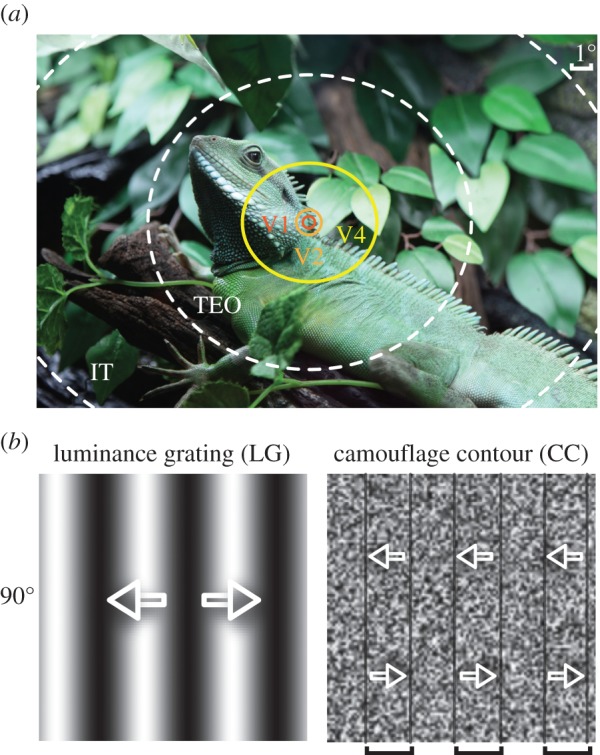
Illustration of different apertures in the sampling of a camouflaged animal in a natural scene and the visual stimuli. (*a*) Superposition of the RFs of neurons from different visual areas on the image of a camouflaged Chinese water dragon (*Physignathus cocincinus*, photo taken in Shanghai aquarium by the author). TEO and IT: inferior temporal areas. (*b*) The LG and CC stimuli with a vertical orientation of 90°. Arrows superimposed represent the bidirectional motion of the global contours of LG and CC stimuli that move leftwards for 2 s and then rightwards for another 2 s. The CCs are indicated by the square brackets and black borders because they disappear as soon as the noise texture stops moving. For simplicity, CC stimuli contain no colour.

Camouflage is a critical evolutionary development for animal survival, as it prevents detection and recognition of both prey and predators in their natural environments (figure 1*a*). For instance, a camouflaged animal such as a jungle lizard or an ocean cuttlefish alters its appearance to closely match its surroundings and in doing so conceals itself from both prey and/or predators [1,2]. Yet as soon as it moves, the camouflage breaks and the animal becomes visible [3,4]. Because of the critical importance of motion to breaking camouflage, many animals have developed complex movement strategies to continuously minimize their motion signals [5,6]. Even when the camouflage breaks, full recognition of a moving animal within its environment remains challenging, i.e. motion aids detection but not necessarily identification [7]. This is most likely owing to the weak contrast or contour disruptions between the moving animal and its adapted-to environment [8]. Therefore, in a natural setting, the motion awareness of a camouflaged animal often comes before the recognition of the animal itself. Camouflaged predators typically approach their prey stealthily before attacking with a burst of high speed. Thus, motion is the first potent segmentation cue for the detection of a camouflaged animal, whose visibility is often correlated with its speed of motion. Furthermore, fast-moving objects generate a spatial orientation code, commonly known as motion steak owing to the temporal integration of the visual system [9–14]. We hypothesize that a camouflage-breaking motion engages motion-streak processing in the primate early visual system when an object moves above a certain speed [14–16].

Human psychophysical studies, inspired by the motion of camouflaged animals in natural scenes, have investigated the perceptual mechanisms underlying second-order or non-luminance defined motion perception [17,18]. These studies posit the existence of a secondary motion processing stage distinct from the primary motion stage for the detection of luminance displacements [19,20]. Physiological studies of neuronal responses from the visual cortices of old-world macaques have focused on the orientation-cue invariance for the processing of various motion-defined boundaries. Eighty-nine per cent (143 out of 160) of units recorded in macaque V1 was found to be activated by luminance and temporal texture bars. However, among all these activated units, only around 21.7% (31 out of 143) of units retained the same orientation preference for both stimuli [21]. As direction-selective cells locate mainly in layer 4B and 6 in macaque V1 [22–24], it is not surprising that less than 10% of V1 cells were direction selective. Interestingly, recent findings from the common marmoset, a New World primate, found approximately 42% (34 out of 81) of V1 cells recorded from displayed invariant responses to stimulus orientation or direction of motion when probed with luminance bars or random-noise bars moving on a flickering background [25].

Neurons in macaque middle temporal (MT) area have much larger receptive fields (RFs) than in V1 and V2 (figure 1*a*) [26,27], and most neurons are selective for dynamic temporal textured bars similar to a subset of V1 neurons [21,28]. Unexpectedly, neurons in macaque MT are not selective to the orientation of static boundaries defined by coherent local motion but instead are thought to encode local motion vectors [29]. The overall percentage of neurons in macaque V4 responding to motion-defined gratings or shapes has also been reported to be quite low (in the range of 10–20%) [30,31]. Single-unit recordings in macaques appear consistent with findings from human brain imaging where no specific visual area has been found to exclusively encode motion-defined boundaries [32–34], although areas V3 and V3A respond to oriented shapes generated by motion-defined stimuli [34]. Further along the visual hierarchy, motion-defined shape is assumed to be fully represented in a cue-invariant manner in the inferior temporal (IT) area [35–37]. This might explain why the recognition of a camouflage-breaking animal occurs slowly compared with the initial processing of the motion that gives the animal away. Surprisingly, no studies have directly compared the change of orientation-selective responses to stimuli mimicking camouflaged movements with low and high speeds in macaque V1 and V2, yet this information is critically important for understanding the breadth of camouflage-breaking motions that occur in nature [6].

In this study, we compared the cortical population responses to moving oriented contours created from sine-wave luminance gratings (LG) to random-noise inducers moving coherently against a static random-noise-textured background. Both types of stimuli had the same orientation and motion direction (figure 1*b*). Such drifting noise gratings, which disappear immediately when they stop moving, were chosen to more accurately mimic the motion of a camouflaged animal against a static background than static motion-defined contours [38] or dynamic contours moving against a dynamic noise background [25]. We found that direction and motion-streak signals within macaque V1 and V2 underlies the processing of camouflage-breaking stimuli at low and high speeds, respectively.

## 2. Material and methods

### (a) Animal preparation and maintenance

Nine adult rhesus macaques (five male and four female) (*Macaca mulatta*) weighing 3.0–5.0 kg were prepared and maintained (see the electronic supplementary material) for optical imaging as previously described [15,39,40].

### (b) Visual stimuli

A gamma-corrected monitor (Sony G520, 1280 × 960 pixels, 100 Hz, luminance ranged from 0.2 to 82 cd m^−2^) was placed 57 cm in front of the animal eyes covering 40 × 30 degrees. Full-screen visual stimuli were generated using Psychtoolbox-3 and MatLab 2010a run under Windows XP and were presented binocularly. We used eight orientations of LG stimuli (figure 1*b*) with spatial frequency (SF) of 1–2 cycles degree^−1^ and temporal frequency of 5 cycles s^−1^. For random-dot stimuli, dot diameter was 0.2°, density 5 dots degree^−2^, and a dot speed of 1–2° s^−1^ (degree per second, with no accumulation). We generated camouflage-breaking stimuli (figure 1*b*), which were moving oriented camouflage contours (CC) created from local random-noise inducers moving coherently against a static random-noise-texture background. The two-dimensional noise texture was composed of randomly positioned noise elements with each element spanning approximately 3.6 arcmin and had a mean luminance of 41 cd m^−2^. The CC SF was 0.375 cycles degree^−1^ and the speeds of CC inducers were 1° s^−1^ or 7° s^−1^ in this experiment. The equations for generating CC stimuli together with flash movie simulations of the stimuli are presented in the electronic supplementary material.

### (c) Optical imaging and data analysis

Intrinsic-signal optical imaging was used as described previously [15,39,40] to measure the cortical response of V1, V2 and V4. Orientation and direction preference maps were classically constructed using a vector summation algorithm [41]. Angular differences between different pairs of orientation and direction preference maps were calculated and histograms were constructed to display the results. Response profile analysis was performed to extract the orientations best represented by the differential orientation maps [15,42,43] (see the electronic supplementary material for details). For the quantification of the response amplitude, the max Δ*R*/*R* values (changes of reflected light) in each responsive patch of a differential map were averaged.

### (d) Model simulation

To simulate the neural responses of V1 and V2 populations, we used a spatio-temporal energy model [15,39,44]. For the simulation responses to camouflage-breaking stimuli, a total of 32 trials were averaged. The responses to two orthogonal stimuli were subtracted to match our optical imaging results. The equations and descriptions of the energy model are detailed in the electronic supplementary material.

## 3. Results

Most neurons in V1 and V2 of macaques are orientation selective with small spatio-temporal RFs and precise retinotopic coordinates [45,46]. These orientation-selective neurons respond invariantly to bidirectional moving gratings or bars and are clustered into columns/domains, from which the population activities (mainly within cortical layers 2/3) can be recorded topographically using intrinsic-signal optical imaging [47,48].

### (a) Camouflage-breaking stimuli activated orientation domains

**Figure 2.**
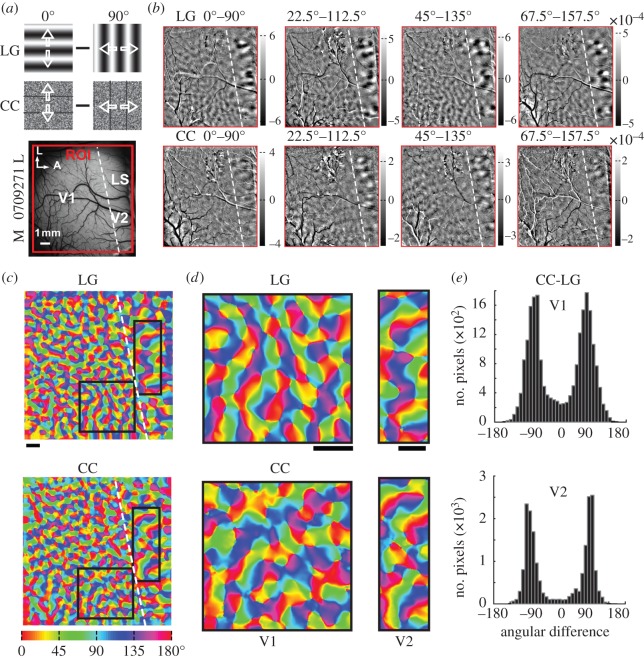
Orientation domains activated by CC stimuli at 7° s^−1^ in V1 and V2. (*a*) Examples of LG and CC stimuli with 0° and 90° orientations and the ROIs in the left hemisphere of macaque 0709271. L, lateral; A, anterior. (*b*) Differential orientation maps for different pairs of LG (top panel) and CC (bottom panel) stimuli. The scale bar of the differential map represents the absolute response amplitude. (*c*) Orientation preference maps of LG and CC of recorded region. (*d*) Representative areas from orientation preference maps of LG and CC in V1 and V2. (*e*) Histograms of angular differences between the two pairs of orientation preference maps of V1 and V2 in (*d*) with peaks around ±90°. The percentages of pixels with preferred orientations shifted by at least 60° amounted to 85% and 91% in V1 and V2, respectively. Scale bar in all panels: 1 mm and the same thereafter.

We generated differential maps of orientation preference by subtracting intrinsic signals evoked by alternating full-field stimuli with orthogonal orientations. We did this for both LG and camouflage-breaking stimuli, moving bidirectionally at 7° s^−1^ (a speed that generates robust cortical responses). Figure 2*a* depicts examples of the 0° and 90° stimuli. In the differential response maps, dark regions prefer the first stimulus condition and bright regions prefer the second (figure 2*b*). Both LG and camouflage-breaking stimuli activated orientation domains within the same region of interest (ROI) of V1 and V2. However, when measuring the relative change in the amount of reflected light (Δ*R*/*R*), response magnitudes elicited by camouflage-breaking stimuli were only about 50% of those activated by LG stimuli (figure 2*b*, see scale bars). These weaker population responses are consistent with the idea that camouflaged movements are harder to detect. We constructed orientation preference maps for the LG and camouflage-breaking stimuli (figure 2*c*,*d*) and found that the angular differences of preferred orientations for camouflage-breaking and LG stimuli peaked around ±90° using pixel-by-pixel subtraction (figure 2*e*).

### (b) Orientation preferences to the camouflage-breaking stimuli dependent on speed

Without exception population responses of V1 and V2 to camouflage-breaking stimuli moving at 7° s^−1^ displayed robust tuning with 90° shifts in orientation preferences compared with LG stimuli (figure 2*e*). A recent study [15] investigating the motion-streak [11,14,49] (where faster texture/dot motion can activate orientation-selective cells with preferences parallel to the motion direction) has shown that full-field noise patterns moving at 7° s^−1^ activated orientation domains with orthogonal preference to those activated at 1° s^−1^ or by moving LGs. We wondered whether the cortical population responses were dependent on the speed of the camouflage-breaking stimuli.

**Figure 3.**
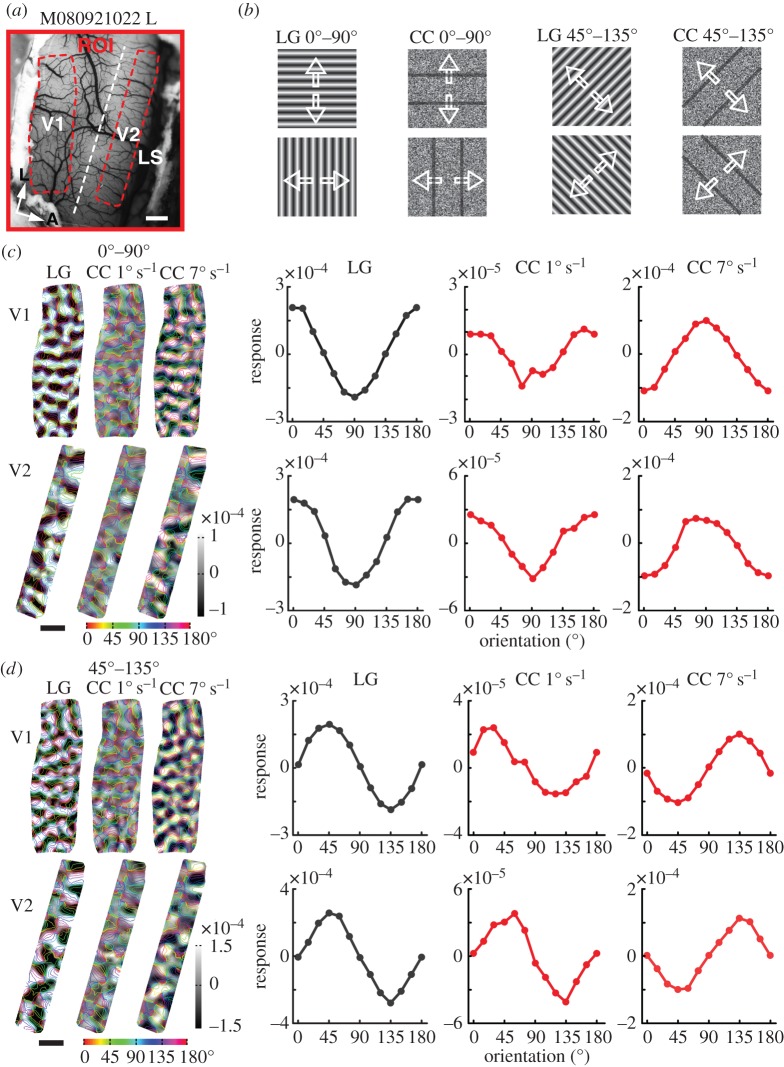
Orientation domains in V1 and V2 activated by CC stimuli at speeds of 1 and 7° s^−1^. (*a*) The surface vasculature of the left hemisphere of macaque 080921022 with ROI of V1 and V2. (*b*) The LG and CC stimulus pairs. (*c*) Differential maps for 0° and 90° orientation pairs from V1 (upper panel) and V2 (lower panel) as areas boxed in (*a*) superimposed with coloured iso-orientation outlines derived from the orientation preference map generated with LG stimuli. The response profiles depict the orientation preferences of CC stimuli moving at speeds of 1 and 7° s^−1^. (*d*) The results for CC stimuli of 45° and 135° orientation pairs moving at speeds of 1 and 7° s^−1^. The responses represent the average pixel values within orientation bins defined by using the LG orientation preference map.

We tested this hypothesis in macaque V1, V2 and V4 by comparing the cortical responses to camouflage-breaking stimuli moving at speeds of 1 and 7° s (figure 3). Within the response regions of V1 and V2 (figure 3*a*), the differential orientation responses to camouflage-breaking stimuli moving forwards and backwards (figure 3*b*) at speeds 1 and 7° s^−1^ were superimposed with coloured iso-orientation contours derived from the orientation preference map generated using LG stimuli (figure 3*c*,*d*). With orientation response profile analysis [42], we found that the orientation preference responses in both V1 and V2 elicited by camouflage-breaking stimuli moving at 1° s^−1^ were in close register to those evoked by LG stimuli, while those activated by camouflage-breaking stimuli moving at 7° s^−1^ were orthogonal, with a roughly 90° shift in orientation preference (tuning curves in figure 3*c*,*d*). Similar results were also observed in V4 (see an example in the electronic supplementary material, figure S1).

### (c) Unidirectional camouflage-breaking stimuli activated direction domains in V2

The majority of neurons in macaque V1, V2 and V4 are orientation selective and respond equivalently to a LG stimulus moving in either of two opposing directions. We thus inferred that at 1° s^−1^, it was the direction of camouflage-breaking stimuli moving forwards and backwards that was responsible for activating the orientation columns as it was with the LG stimuli. In order to confirm this, we asked whether the unidirectional motion of the camouflage-breaking stimuli at 1° s^−1^ could activate direction-selective domains in macaque V2. No direction-selective domains can be mapped in macaque V1 by using intrinsic optical imaging [15,50]. We used full-field random dots moving in four opposite directions (0°, 180°, 90° and 270°) at a speed of 1° s^−1^ as our control stimuli (electronic supplementary material, figure S2*a*,*b*). Camouflage-breaking stimuli moving at a speed of 1° s^−1^, activated direction domains with similar response patterns in the same locations of V2 as the random dots (electronic supplementary material, figure S2*c*). Pixel-by-pixel subtraction of the angles of preferred directions for random dots and camouflage-breaking stimuli showed histograms peaking around 0° for both ROIs (electronic supplementary material, figure S2*d*). Similar results were observed in all macaques tested. These findings demonstrate that unidirectional signals in camouflage-breaking stimuli moving at 1° s^−1^ generate responses from direction columns in segregated locations of V2.

### (d) Local motion cues responsible for the population responses

**Figure 4.**
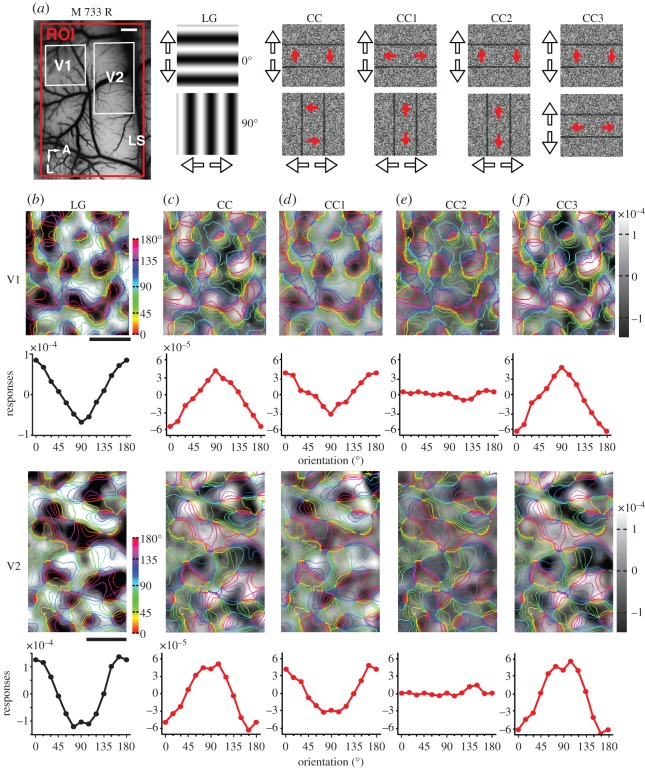
Orientation domains activated by different variations of CC stimuli in V1 and V2. (*a*) Picture of the surface vasculature taken from the right hemisphere of macaque 733 with ROI indicated by red box. White boxes denote regions of V1 and V2 that were examined in detail. Cartoons of the different stimulus pairs are illustrated. Arrows outside the panels (black outline) indicate the bidirectional motion of the luminance or camouflage contours. Arrows superimposed on the panels (red filled) indicate the axis of motion of the coherent noise inducers. (*b*–*f*) Differential orientation preference maps with colour coded iso-orientation contours superimposed and orientation response profiles derived from stimulus pairs depicted in (*a*).

As shown above, the population responses to camouflage-breaking stimuli are dependent on motion speed. We reasoned that it must be the motion of the local-noise inducers, not the global camouflage contours, that activates the orientation domains in V1 and V2. This idea is outlined in the electronic supplementary material, figure S3. Motion-defined contours can be generated by noise moving in any direction [19,20]. So we designed different variants (CC1–5) of the camouflage-breaking stimuli to test whether it was the local motion cues that dominated the cortical responses. We analysed the responses of orientation domains from the same locations in V1 and V2 when the camouflage contours were induced by noise moving either parallel or perpendicular to the contour orientations (figure 4*a*).

In our standard camouflage-breaking stimuli, the direction of coherent noise motion was perpendicular to the orientation of the camouflage contours. Consistent with the results in figures 2 and 3, the preferences of orientation domains activated by the LG and camouflage-breaking stimuli moving at 7° s^−1^ were orthogonal in both V1 and V2 (figure 4*b*,*c*; LG versus CC). For the CC1 variant, the global camouflage contour moved identically to the camouflage-breaking stimuli, but the direction of the local noise inducers was parallel rather than perpendicular to the orientation of the camouflage contours. The pattern of orientation domains activated by CC1 was closely in register with that elicited by LG in both V1 and V2 (figure 4*b*,*d*; LG versus CC1). CC2 contained exactly the same horizontal and vertical global camouflage contours as CC and CC1; however, in this case, the local noise inducers of the two stimulus conditions moved along an identical axis of motion (perpendicular to the horizontal camouflage contours and parallel to the vertical camouflage contours). This configuration contained the same local motion cues in both cases (that move in the same direction) and failed to produce any responsive domains in either V1 or V2, resulting in an almost flat tuning curve (figure 4*e*; CC2) despite the fact that global camouflage contours with orthogonal orientations were used. In our last configuration, CC3, population responses were activated by global camouflage contours of the same orientation, but with local noise motion set at right angles to each other (i.e. the same orthogonal axes of motion as in CC). Once again, reversed orientation preference maps emerged from responses in both V1 and V2 (figure 4*b*,*f*; LG versus CC3). Electronic supplementary material, figure S4, shows the results from another macaque, where the noise moved at an acute angle to the camouflage contours (CC4 and CC5). Similar findings were observed in all animals studied. Together, these findings demonstrate that it was the motion of the local-noise inducers, rather than the orientation of the global camouflage contours, that was responsible for the activation of orientation domains in V1 and V2.

### (e) A spatio-temporal energy filter model simulated all population responses

Recently, V1 population responses across species [15,44,51,52] have been successfully reproduced by a spatio-temporal linear filter model. We implemented this generic energy model to systematically simulate the population responses in macaque V1 and V2 to camouflage-breaking stimuli at high and low speeds. We used the camouflage-breaking stimuli of 0° and 90° orientations moving in eight opposing directions at 7° s^−1^ to simulate V1 and V2 population responses (electronic supplementary material, figure S5*a*). The orientation response profiles derived from the model simulation closely matched those in our experiments. Specifically, the resulting response profiles were reversed for CC and CC1 stimuli (as in figure 4*c*,*d*) and also for CC4 and CC5 stimuli (as in electronic supplementary material, figure S4). We further simulated V1 and V2 population responses to camouflage-breaking stimuli at 1° s^−1^ and the resulting simulated orientation response profiles were reversed to those derived from the camouflage-breaking stimuli moving at 7° s^−1^ (electronic supplementary material, figure S5*b*), consistent with the observations revealed by optical imaging.

In both V1 and V2, the simulated response strength for LG stimuli was the highest, while the simulated response strength for camouflage-breaking stimuli moving at 1° s^−1^ was the lowest and that for camouflage-breaking stimuli moving at 7° s^−1^ was in the middle (electronic supplementary material, figure S5c). These simulations closely matched the trend of the average response strengths directly measured in the optical imaging experiments (electronic supplementary material, figure S5*d*). We found that the average population responses to LG stimuli were significantly larger than those to camouflage-breaking stimuli moving at speed 7° s^−1^ in both V1 and V2 (*p* < 0.05, *n* = 12, paired *t*-test). In addition, the responses to camouflage-breaking moving at speed 1° s^−1^ were significantly weaker than those to camouflage-breaking stimuli moving at speed 7° s^−1^ (*p* < 0.05, *n* = 12, paired *t*-test). Together, these results suggest that the population responses of V1 and V2 to camouflage-breaking stimuli can be accounted for by a simple linear spatio-temporal filter mechanism.

## 4. Discussion

Animal camouflage is a striking example of a natural evolutionary structural and behavioural adaptation that has long fascinated many branches of scientific investigation [8]. How the visual brain detects camouflaged animal movements and subsequently recognizes the camouflaged animal remains unclear. This study explores the first part of this question by examining population responses across visual areas in macaques, the best animal model for human vision. We found that orientation-selective domains within macaque V1, V2 and V4 engaged in the processing of different components of camouflage-breaking motion at low and high speeds.

### (a) The population responses of macaque V1, V2 and V4 to camouflage-breaking stimuli

In macaques, most direction-selective neurons in V1 are simple or complex cells residing in layers 4B and 6 [22,23,46]. The neurons in these layers are too deep for their activity to be recorded by intrinsic-signal optical imaging. As the majority of neurons in V1 layers 2/3 are orientation-selective and respond equally well to motion in opposite directions, it was the noise inducers moving bidirectionally at 1° s^−1^ that were responsible for the activation of orientation domains in V1 and thereafter in V2 and V4. However, as previously reported, when the speed of motion was increased to 7° s^−1^, the movement of the random-noise texture generated a spatial orientation signal (commonly referred to as the motion-streak) parallel to the motion direction of the moving noise stimuli [15]. Thus, the orientation-selective neurons with preferences parallel to the motion streak were activated and their responses were subsequently recorded by intrinsic optical imaging as orientation domains in V1, V2 and V4. The temporal integration of visual inputs along the axis of motion was also precisely accounted for by the spatio-temporal energy model, consistent with the notion that motion can be linearly processed in primate early visual cortices [15,53,54]. In comparison to LG stimuli, the weaker population responses to camouflage-breaking stimuli may relate to the poor visibility of concealed animals even when moving, supporting the notion that motion cannot entirely ‘break’ camouflage owing to closely matched appearances between the camouflaged animal and its adapted-to environment [5–7,55,56].

### (b) Neural responses of macaque V1, V2 and V4 to global camouflage contours

Single-unit recordings in New World marmosets have revealed that orientation tuned neural responses to moving camouflaged bars are prevalent in the MT area [57] and are also common in early visual cortices [25,58]. An previous study reported that less than a quarter of all activated cells (31 out of 143) in macaque V1 were selective to the orientation of both camouflage bars and LG [21]. However, neither of these V1 studies explicitly investigated the change of orientation selectivity with the speed of motion of the camouflaged bars. Bourne *et al.* [25] classified 42% of their units as cue-invariant meaning that either the direction selectivity or orientation selectivity of the cell remained similar (less than 30° difference) for both luminance-defined and noise-defined bars. They used a range of stimulus speeds (from 7–86° s^−1^) and did not report any change in selectivity with speed (i.e. motion-streak effects). Their findings seem to contradict previous studies that have examined the phenomenon of motion streak using random dots and noise patterns [15,16]. Perhaps the presence of a twinkling background, in the Bourne *et al.* [25] study, made it impossible for the motion-steak signal to develop and so interfered with the orientation selectivity.

Another study reported even a lower percentage of cells (less than 10%) in macaque V1 and V2 showing cue-invariant responses to the orientation of motion-defined but static contours [38], although this study also did not look at the effect of motion streak. As intrinsic signals are derived from pooled neuronal activities, it is unlikely that intrinsic optical imaging would reveal this small percentage of cells in macaque V1 and V2; and even in V4 where the number of neurons responding to motion-defined contours and shapes is reported to be higher (up to around 20%) [30,31]. Hence, the population response in V1, V2 and V4 mainly encodes the local motion signals in camouflage-breaking stimuli as revealed here both experimentally and mathematically. A very recent study in awake macaques has however claimed that these small number of neurons in macaque V2 but not V1 can be recorded by intrinsic optical imaging, and that motion-defined boundaries are thus mapped in orientation domains of V2 [59]. These authors used static boundaries defined by dot motion fields moving at slow speeds (1 to 2° s^−1^) and the motion axis of the moving dots was always at a 45° angle with respect to the motion defined strip borders. Unfortunately, they did not test their stimuli at higher speeds and did not vary the local motion direction of the random dots relative to the global motion-defined boundary orientation. Hence, it is uncertain if their motion boundary results are invariant or not to speed as well as to local inducer motion direction. In both V1 and V2 of anesthetized macques as we have demonstrated, it is the movement of the local-noise inducers within our camouflage-breaking stimuli that dominates the neural responses we recorded. However, neurons encoding motion-defined contours were prevalent in cat visual area 18 [60], suggesting carnivores may have developed more efficient ways for detecting camouflaged movements.

It is expected that neurons in MT, the motion centre of primates which receives direction signal inputs from V1 layers 4B/6, V2 thick strips, and directly from the thalamus [22–24,61], will also encode motion direction/axis signals within camouflaged motion. Furthermore, as a higher percentage of cells in both the V3 and IT area have been found to respond to simple geometric shapes defined by motion-defined contours [31,32,34–37], it is more likely that robust responses to motion-defined contours as well as to camouflage contours will be found in these higher visual areas. Thus, the recognition of a camouflaged animal on the move must engage multiple visual areas working together.

## 5. Concluding remarks

A thought-provoking implication of our findings relates to the wide prevalence of conspicuous oriented stripes and zig-zags found across many animal species (for example zebras) and used by humans in camouflage designed for moving objects (famously warships in World War I and II). These oriented-stripe patterns, though more salient when static, are hypothesized to generate a ‘motion dazzle’ that makes it difficult for either predator or prey to judge speed and/or direction of motion [8]. In addition, the interference by oriented motion dazzle has been observed in human psychophysical tests, where oriented patterns can impede motion task performance [62–64], and/or cause misperceptions of speed [65,66]. Thus, a more detailed understanding of the neural population responses to direction and motion-streak signals across speeds can usefully constrain and inform future behavioural studies of motion crypsis. In summary, low-speed camouflage-breaking movements are encoded by both direction- and orientation-selective cells corresponding to the direction of motion of the concealed animal, while high-speed camouflage-breaking movements generate a motion-streak signal that is encoded by a complementary set of orientation-selective cells within V1, V2 and V4 of the macaque ventral visual stream.

## Supplementary Material

ESM for Yin et al July 06 2015

## Supplementary Material

EnergyModelforCamouflageBreakingMotion

## Data accessibility

This paper does not contain online raw data.

## References

[1] Kelman EJ, Osorio D, Baddeley RJ 2008 A review of cuttlefish camouflage and object recognition and evidence for depth perception. J. Exp. Biol. 211, 1757–1763. (10.1242/jeb.015149) 18490391

[2] Stevens M, Merilaita S 2011 Animal camouflage: from mechanisms to function. Cambridge, UK: Cambridge University Press.

[3] Regan D, Beverley KI 1984 Figure-ground segregation by motion contrast and by luminance contrast. J. Opt. Soc. Am. A 1, 433–442. (10.1364/JOSAA.1.000433) 6726491

[4] Zylinski S, Osorio D, Shohet AJ 2009 Perception of edges and visual texture in the camouflage of the common cuttlefish, *Sepia officinalis*. Phil. Trans. R. Soc. B 364, 439–448. (10.1098/rstb.2008.0264) 18990667 PMC2674086

[5] Srinivasan MV, Davey M 1995 Strategies for active camouflage of motion. Proc. R. Soc. Lond. B 259, 3029–3031. (10.1098/rspb.1995.0004)

[6] Troscianko T, Benton CP, Lovell PG, Tolhurst DJ, Pizlo Z 2009 Camouflage and visual perception. Phil. Trans. R. Soc. B 364, 449–461. (10.1098/rstb.2008.0218) 18990671 PMC2674079

[7] Hall JR, Cuthill IC, Baddeley R, Shohet AJ, Scott-Samuel NE 2013 Camouflage, detection and identification of moving targets. Proc. R. Soc. B 280, 20130064 (10.1098/rspb.2013.0064) PMC3619462 23486439

[8] Stevens M, Merilaita S 2009 Animal camouflage: current issues and new perspectives. Phil. Trans. R. Soc. B 364, 423–427. (10.1098/rstb.2008.0217) 18990674 PMC2674078

[9] Alais D, Apthorp D, Karmann A, Cass J 2011 Temporal integration of movement: the time-course of motion streaks revealed by masking. PLoS ONE 6, e28675 (10.1371/journal.pone.0028675) 22205961 PMC3243686

[10] Apthorp D, Schwarzkopf DS, Kaul C, Bahrami B, Alais D, Rees G 2013 Direct evidence for encoding of motion streaks in human visual cortex. Proc. R. Soc. B 280, 20122339 (10.1098/rspb.2012.2339) PMC3574303 23222445

[11] Burr DC, Ross J 2002 Direct evidence that ‘speedlines’ influence motion mechanisms. J. Neurosci. 22, 8661–8664. 12351740 10.1523/JNEUROSCI.22-19-08661.2002 PMC6757803

[12] Edwards M, Crane MF 2007 Motion streaks improve motion detection. Vis. Res. 47, 828–833. (10.1016/j.visres.2006.12.005) 17258262

[13] Francis G, Kim H 2001 Perceived motion in orientational afterimages: direction and speed. Vis. Res. 41, 161–172. (10.1016/S0042-6989(00)00242-X) 11163851

[14] Geisler WS 1999 Motion streaks provide a spatial code for motion direction. Nature 400, 65–69. (10.1038/21886) 10403249

[15] An X, Gong H, Qian L, Wang X, Pan Y, Zhang X, Yang Y, Wang W 2012 Distinct functional organizations for processing different motion signals in V1, V2, and V4 of macaque. J. Neurosci. 32, 13 363–13 379. (10.1523/JNEUROSCI.1900-12.2012) PMC6621371 23015427

[16] Geisler WS, Albrecht DG, Crane AM, Stern L 2001 Motion direction signals in the primary visual cortex of cat and monkey. Vis. Neurosci. 18, 501–516. (10.1017/S0952523801184014) 11829297

[17] Cavanagh P, Mather G 1989 Motion: the long and short of it. Spat. Vis. 2, 103–129. (10.1163/156856889X00077) 2487159

[18] Chubb C, Sperling G 1988 Drift-balanced random stimuli: a general basis for studying non-Fourier motion perception. J. Opt. Soc. Am. A 5, 1986–2007. (10.1364/JOSAA.5.001986) 3210090

[19] Zanker JM 1993 Theta motion: a paradoxical stimulus to explore higher order motion extraction. Vis. Res. 33, 553–569. (10.1016/0042-6989(93)90258-X) 8503201

[20] Zanker JM, Hupgens IS 1994 Interaction between primary and secondary mechanisms in human motion perception. Vis. Res. 34, 1255–1266. (10.1016/0042-6989(94)90201-1) 8023435

[21] Chaudhuri A, Albright TD 1997 Neuronal responses to edges defined by luminance vs. temporal texture in macaque area V1. Vis. Neurosci. 14, 949–962. (10.1017/S0952523800011664) 9364731

[22] Hawken MJ, Parker AJ, Lund JS 1988 Laminar organization and contrast sensitivity of direction-selective cells in the striate cortex of the Old World monkey. J. Neurosci. 8, 3541–3548. 3193169 10.1523/JNEUROSCI.08-10-03541.1988 PMC6569616

[23] Movshon JA, Newsome WT 1996 Visual response properties of striate cortical neurons projecting to area MT in macaque monkeys. J. Neurosci. 16, 7733–7741. 8922429 10.1523/JNEUROSCI.16-23-07733.1996 PMC6579106

[24] Nassi JJ, Callaway EM 2009 Parallel processing strategies of the primate visual system. Nat. Rev. Neurosci. 10, 360–372. (10.1038/nrn2619) 19352403 PMC2771435

[25] Bourne JA, Tweedale R, Rosa MG 2002 Physiological responses of New World monkey V1 neurons to stimuli defined by coherent motion. Cereb. Cortex 12, 1132–1145. (10.1093/cercor/12.11.1132) 12379602

[26] Gattass R, Gross CG 1981 Visual topography of striate projection zone (MT) in posterior superior temporal sulcus of the macaque. J. Neurophysiol. 46, 621–638. 7299437 10.1152/jn.1981.46.3.621

[27] Rousselet GA, Thorpe SJ, Fabre-Thorpe M 2004 How parallel is visual processing in the ventral pathway? Trends Cogn. Sci. 8, 363–370. (10.1016/j.tics.2004.06.003) 15335463

[28] Albright TD 1992 Form-cue invariant motion processing in primate visual cortex. Science 255, 1141–1143. (10.1126/science.1546317) 1546317

[29] Marcar VL, Xiao DK, Raiguel SE, Maes H, Orban GA 1995 Processing of kinetically defined boundaries in the cortical motion area MT of the macaque monkey. J. Neurophysiol. 74, 1258–1270. 7500149 10.1152/jn.1995.74.3.1258

[30] Mysore SG, Vogels R, Raiguel SE, Orban GA 2006 Processing of kinetic boundaries in macaque V4. J. Neurophysiol. 95, 1864–1880. (10.1152/jn.00627.2005) 16267116

[31] Mysore SG, Vogels R, Raiguel SE, Orban GA 2008 Shape selectivity for camouflage-breaking dynamic stimuli in dorsal V4 neurons. Cereb. Cortex 18, 1429–1443. (10.1093/cercor/bhm176) 17934186

[32] Larsson J, Heeger DJ, Landy MS 2010 Orientation selectivity of motion-boundary responses in human visual cortex. J. Neurophysiol. 104, 2940–2950. (10.1152/jn.00400.2010) 20861432 PMC3007646

[33] Seiffert AE, Somers DC, Dale AM, Tootell RB 2003 Functional MRI studies of human visual motion perception: texture, luminance, attention and after-effects. Cereb. Cortex 13, 340–349. (10.1093/cercor/13.4.340) 12631563

[34] Zeki S, Perry RJ, Bartels A 2003 The processing of kinetic contours in the brain. Cereb. Cortex 13, 189–202. (10.1093/cercor/13.2.189) 12507950

[35] Orban GA 2008 Higher order visual processing in macaque extrastriate cortex. Physiol. Rev. 88, 59–89. (10.1152/physrev.00008.2007) 18195083

[36] Sary G, Vogels R, Kovacs G, Orban GA 1995 Responses of monkey inferior temporal neurons to luminance-defined, motion-defined, and texture-defined gratings. J. Neurophysiol. 73, 1341–1354. 7643152 10.1152/jn.1995.73.4.1341

[37] Sary G, Vogels R, Orban GA 1993 Cue-invariant shape selectivity of macaque inferior temporal neurons. Science 260, 995–997. (10.1126/science.8493538) 8493538

[38] Marcar V, Raiguel S, Xiao D, Orban G 2000 Processing of kinetically defined boundaries in areas V1 and V2 of the macaque monkey. J. Neurophysiol. 84, 2786–2798. 11110809 10.1152/jn.2000.84.6.2786

[39] An X et al. 2014 Orientation-cue invariant population responses to contrast-modulated and phase-reversed contour stimuli in macaque V1 and V2. PLoS ONE 9, e106753 (10.1371/journal.pone.0106753) 25188576 PMC4154761

[40] Pan Y, Chen M, Yin J, An X, Zhang X, Lu Y, Gong H, Li W, Wang W 2012 Equivalent representation of real and illusory contours in macaque V4. J. Neurosci. 32, 6760–6770. (10.1523/JNEUROSCI.6140-11.2012) 22593046 PMC6622189

[41] Bonhoeffer T, Grinvald A 1996 Optical imaging based on intrinsic signals: the methodology. In Brain Mapping: the methods (eds Toga AW, Mazziotta JC), pp. 55–97. San Diego, CA: Academic Press.

[42] Basole A, White LE, Fitzpatrick D 2003 Mapping multiple features in the population response of visual cortex. Nature 423, 986–990. (10.1038/nature01721) 12827202

[43] Zhan CA, Baker CL Jr 2006 Boundary cue invariance in cortical orientation maps. Cereb. Cortex 16, 896–906. (10.1093/cercor/bhj033) 16151176

[44] Mante V, Carandini M 2005 Mapping of stimulus energy in primary visual cortex. J. Neurophysiol. 94, 788–798. (10.1152/jn.01094.2004) 15758051

[45] De Valois RL, Yund EW, Hepler N 1982 The orientation and direction selectivity of cells in macaque visual cortex. Vis. Res. 22, 531–544. (10.1016/0042-6989(82)90112-2) 7112953

[46] Hubel DH, Wiesel TN 1968 Receptive fields and functional architecture of monkey striate cortex. J. Physiol. 195, 215–243. (10.1113/jphysiol.1968.sp008455) 4966457 PMC1557912

[47] Blasdel GG 1992 Differential imaging of ocular dominance and orientation selectivity in monkey striate cortex. J. Neurosci. 12, 3115–3138. 1494950 10.1523/JNEUROSCI.12-08-03115.1992 PMC6575665

[48] Ts'o DY, Frostig RD, Lieke EE, Grinvald A 1990 Functional organization of primate visual cortex revealed by high resolution optical imaging. Science 249, 417–420. (10.1126/science.2165630) 2165630

[49] Jancke D 2000 Orientation formed by a spot's trajectory: a two-dimensional population approach in primary visual cortex. J. Neurosci. 20, RC86. 10875941 10.1523/JNEUROSCI.20-14-j0005.2000 PMC6772319

[50] Lu HD, Chen G, Tanigawa T, Roe AW 2010 A motion direction map in macaque V2. Neuron 68, 1002–1013. (10.1016/j.neuron.2010.11.020) 21145011 PMC3391546

[51] An X, Gong H, McLoughlin N, Yang Y, Wang W 2014 The mechanism for processing random-dot motion at various speeds in early visual cortices. PLoS ONE 9, e93115 (10.1371/journal.pone.0093115) 24682033 PMC3969330

[52] Baker TI, Issa NP 2005 Cortical maps of separable tuning properties predict population responses to complex visual stimuli. J. Neurophysiol. 94, 775–787. (10.1152/jn.01093.2004) 15758052

[53] De Valois RL, Cottaris NP, Mahon LE, Elfar SD, Wilson JA 2000 Spatial and temporal receptive fields of geniculate and cortical cells and directional selectivity. Vis. Res. 40, 3685–3702. (10.1016/S0042-6989(00)00210-8) 11090662

[54] Rust NC, Schwartz O, Movshon JA, Simoncelli EP 2005 Spatiotemporal elements of macaque V1 receptive fields. Neuron 46, 945–956. (10.1016/j.neuron.2005.05.021) 15953422

[55] Mizutani A, Chahl JS, Srinivasan MV 2003 Insect behaviour: motion camouflage in dragonflies. Nature 423, 604 (10.1038/423604a) 12789327

[56] Zylinski S, How MJ, Osorio D, Hanlon RT, Marshall NJ 2011 To be seen or to hide: visual characteristics of body patterns for camouflage and communication in the Australian giant cuttlefish *Sepia apama*. Am. Nat. 177, 681–690. (10.1086/659626) 21508613

[57] Lui LL, Dobiecki AE, Bourne JA, Rosa MG 2012 Breaking camouflage: responses of neurons in the middle temporal area to stimuli defined by coherent motion. Eur. J. Neurosci. 36, 2063–2076. (10.1111/j.1460-9568.2012.08121.x) 22591321

[58] Lui LL, Bourne JA, Rosa MG 2005 Single-unit responses to kinetic stimuli in New World monkey area V2: physiological characteristics of cue-invariant neurones. Exp. Brain. Res. 162, 100–108. (10.1007/s00221-004-2113-9) 15517211

[59] Chen M, Li P, Zhu S, Han C, Xu H, Fang Y, Hu J, Roe AW, Lu HD In press An orientation map for motion boundaries in macaque V2. Cereb. Cortex. (10.1093/cercor/bhu235) PMC5006290 25260703

[60] Gharat A, Baker CL Jr 2012 Motion-defined contour processing in the early visual cortex. J. Neurophysiol. 108, 1228–1243. (10.1152/jn.00840.2011) 22673328

[61] Shipp S, Zeki S 1989 The organization of connections between areas V5 and V2 in macaque monkey visual cortex. Eur. J. Neurosci. 1, 333–354. (10.1111/j.1460-9568.1989.tb00799.x) 12106143

[62] How MJ, Zanker JM 2014 Motion camouflage induced by zebra stripes. Zoology 117, 163–170. (10.1016/j.zool.2013.10.004) 24368147

[63] Hughes AE, Troscianko J, Stevens M 2014 Motion dazzle and the effects of target patterning on capture success. BMC Evol. Biol. 14, 201 (10.1186/s12862-014-0201-4) 25213150 PMC4172783

[64] Stevens M, Searle WT, Seymour JE, Marshall KL, Ruxton GD 2011 Motion dazzle and camouflage as distinct anti-predator defenses. BMC Biol. 9, 81 (10.1186/1741-7007-9-81) 22117898 PMC3257203

[65] Burr D 2000 Motion vision: are ‘speed lines’ used in human visual motion? Curr. Biol. 10, R440–R443. (10.1016/S0960-9822(00)00545-5) 10873792

[66] von Helversen B, Schooler LJ, Czienskowski U 2013 Are stripes beneficial? Dazzle camouflage influences perceived speed and hit rates. PLoS ONE 8, e61173 (10.1371/journal.pone.0061173) 23637795 PMC3634837

