# Supplementary material for: Breaking cover: neural responses to slow and fast camouflage-breaking motion
Source: Proc Biol Sci. 2015 Aug 22;282(1813):20151182. doi: 10.1098/rspb.2015.1182 (PMC4632627; doi:10.1098/rspb.2015.1182)
Supplement: ESM for Yin et al July 06 2015 [file rspb20151182supp1.pdf]

This file contains Supplementary Materials and Figures with legends for:

Title: Breaking cover: neural responses to slow and fast camouflage-breaking motion

Running title: Visual responses to camouflaged motion

Authors: Jiapeng Yin[1], Hongliang Gong[1], Xu An[1], Zheyuan Chen[1], Yiliang Lu[1*], Ian Andolina[1], Niall McLoughlin[2] and Wei Wang[1*]

1 Institute of Neuroscience, State Key Laboratory of Neuroscience and Key Laboratory of Primate Neurobiology, Shanghai Institutes for Biological Sciences, Chinese Academy of Sciences, Shanghai 200031, P. R. China

2 Faculty of Life Science, University of Manchester, Manchester M13 9PT, UK

1. Materials and methods

Animal preparation and maintenance

Detailed animal preparation and maintenance for intrinsic-signal optical imaging were as as previously described (An et al. 2012; An et al. 2014b; Pan et al. 2012). In brief, the general anesthesia was maintained using propofol (4–5mg•kg^{-1}•h^{-1}, i.v.). Depth of anesthesia was verified with respect to continuously monitored electrocardiogram (ECG), pulse oximeter (SpO_2), and end-tidal carbon dioxide (CO_2). Craniotomy and durotomy were performed on both hemispheres over macaque V1, V2, and V4 for dual optical imaging using two stainless steel chambers of 30 mm diameter secured to skull. The lunate sulcus (LS) and superior temporal sulcus (STS) were used as cortical landmarks for surgeries and imaging. Gas-permeable contact lenses were applied to protect the animal's corneas and the eyes were refracted to focus on the CRT monitor.

Visual stimuli

The camouflage-breaking stimuli can be expressed as:

$$CC(x,y,t) = \frac{\operatorname{sgn}(S(x,y,t))+1}{2}(l_0 + l_0 N_1(x,y)) - \qquad (1)$$

$$\frac{\operatorname{sgn}(S(x,y,t))-1}{2}(l_0 + l_0 N_2(x + \frac{v\sin(\theta)t}{f}, y + \frac{v\cos(\theta)t}{f}))$$

$$S(x,y,t) = \sin(2\pi f(\sin(\theta)x + \cos(\theta)y) + 2\pi vt) \qquad (2)$$

where f is the spatial frequency of CC contours (0.375 cycles per degree in this experiment), θ is the orientation, and v is the temporal frequency of the inducers of camouflage-breaking stimuli (7°/s or 1°/s). $N(x,y)$ is the uniform distribution (-1 to 1) two-dimensional noise texture, which is composed of randomly positioned noise elements with each element spanning approximately 3.6 arcmin. l_0 is the mean luminance (41 cd•m^{-2} in the experiment). We tested camouflaged contour (CC) stimuli with local motion either perpendicular or differing by ±45°.

Optical imaging and data analysis

The boundary of V1 and V2 was defined using ocular dominance mapping, which produced stripe-like compartments perpendicular to the border between V1 and V2 (Blasdel 1992; Lu et al. 2010; Ts'o et al. 1990). The cortical locus (prelunate gyrus) located between LS and STS and 35mm lateral from the midline was selected as a representative area of V4.

For LG stimuli, data were typically averaged over 32 or 64 trials, while for random dots and CC stimuli the data were often averaged over 128 trials. For each trial, the recording period lasted for 21 s, including 1 s prior to the stimulus onset and a stimulus duration of 4 s. Video frames taken after the stimulus onset for 3–7 s were averaged within a region of interest (ROI), and then subtracted and divided by a pure blank (average of prestimulus images) to generate a single-condition map of reflectance change (ΔR/R). The images were high-pass filtered (1.1-1.2 mm in diameter) and smoothed (106-306 μm in diameter) by circular averaging filters to suppress low- and high-frequency noise while avoiding signal distortion. Blood vessel and other noise artifacts were masked based on variability maps which find pixels with large cross-trial variability. Pixels within the mask were never used in quantitative analysis.

The intrinsic-signal strengths were calculated as the relative change in the amount of light reflected (ΔR/R) from the ROIs in V1, V2, and V4 for each stimulus type. For response profile analysis (Supplementary Figure 6A, B)(Basole et al., 2003), the cortical area to be analyzed was divided up into 12 equally spaced orientation bins in a 0 to 180° range according to the orientation preference map (the color map) derived from the responses to LG. The overall pixel values (intensity) within each of the orientation bins were averaged to give a measurement for that orientation of either LG or CC stimulus (Figure 3). While for the quantification of the absolute cortical response amplitude, the max ΔR/R values (the peak response values) in each responsive patch or domain of a differential map (the gray image) were averaged (Supplementary Figure 6C), and this peak response strength was taken as the absolute response amplitude (Supplementary Figure 5D).

Model simulation

V1 and V2 populations were modeled as a bank of spatio-temporal filters. The spatial and temporal filtering properties of neurons were modeled as Gaussian functions:

$$S(p_x, p_y) = \exp(-\frac{[p_x - S_p \sin(\phi)]^2 + [p_y - S_p \cos(\phi)]^2}{2\sigma_S^2}) / (2\pi\sigma_S^2) \quad (3)$$

$$T(\rho) = \exp(-\frac{(\rho - T_\rho)^2}{2\sigma_T^2}) / (\sqrt{2\pi}\sigma_T) \quad (4)$$

where p_x and p_y are spatial frequency coordinates and ρ is temporal frequency coordinates. S_p and T_p are the preferred spatial and temporal frequency. σ_S and σ_T are the spatial and temporal bandwidth. ϕ is the preferred orientation. The V1 or V2 neurons with the same spatial frequency, temporal frequency, and orientation preferences integrate stimulus energy over a small receptive field in frequency space. This can be modeled as:

$$R = \int_0^\infty \int_0^\infty \int_0^\infty \mu_S \mu_T A(p_x, p_y, \rho) S(p_x, p_y) T(\rho) dp_x dp_y d\rho \quad (5)$$

where A is the Fourier transformed stimulus. Only a 15 by 15 degree sub-region of stimuli were used to reduce computation time. *S* and *T* are the proportion of neurons that prefer the specific spatial and temporal frequencies, respectively. Then the responses of a set of neurons that have different orientation, spatial, and temporal frequency tuning properties were averaged. The Matlab code for the energy model is submitted as a Supplementary file online (with file name: EnergyModelforCamouflageBreakingMotion.txt). A 15×15 degree

sub-region of stimuli was used to reduce computation time. The neurons' preferred orientations (25 values) were uniformly distributed over 180°. SFs (14 values) were logarithmically spaced between 0.13–11cpd (mean: 2.2cpd) for V1 (Foster et al. 1985) and 1.4 cpd for V2 (Levitt et al. 1994), following a Gaussian distribution. TFs (16 values) were also logarithmically spaced between 0.25–45Hz (mean: 3.7Hz) for V1 and 3.5Hz for V2 (Foster et al. 1985).

2. Supplementary Flash movies illustrate the camouflage-breaking motion stimuli

Supplementary Movie S1: drafting luminance gratings (LG)

Supplementary Movie S2: camouflaged contour within drafting camouflage-breaking stimuli (CC)

Supplementary Movie S3: camouflaged contour within drafting camouflage-breaking stimuli (CC1)

Supplementary Movie S4: camouflaged contour within drafting camouflage-breaking stimuli (CC2)

Supplementary Movie S5: camouflaged contour within drafting camouflage-breaking stimuli (CC3)

These movies are produced by MATLAB with low resolution for illustration only and the speed for all the movies is 7deg/sec. These files can be opened with Windows Media Player.

3. Supplementary Figures and legends

Supplementary Figure 1

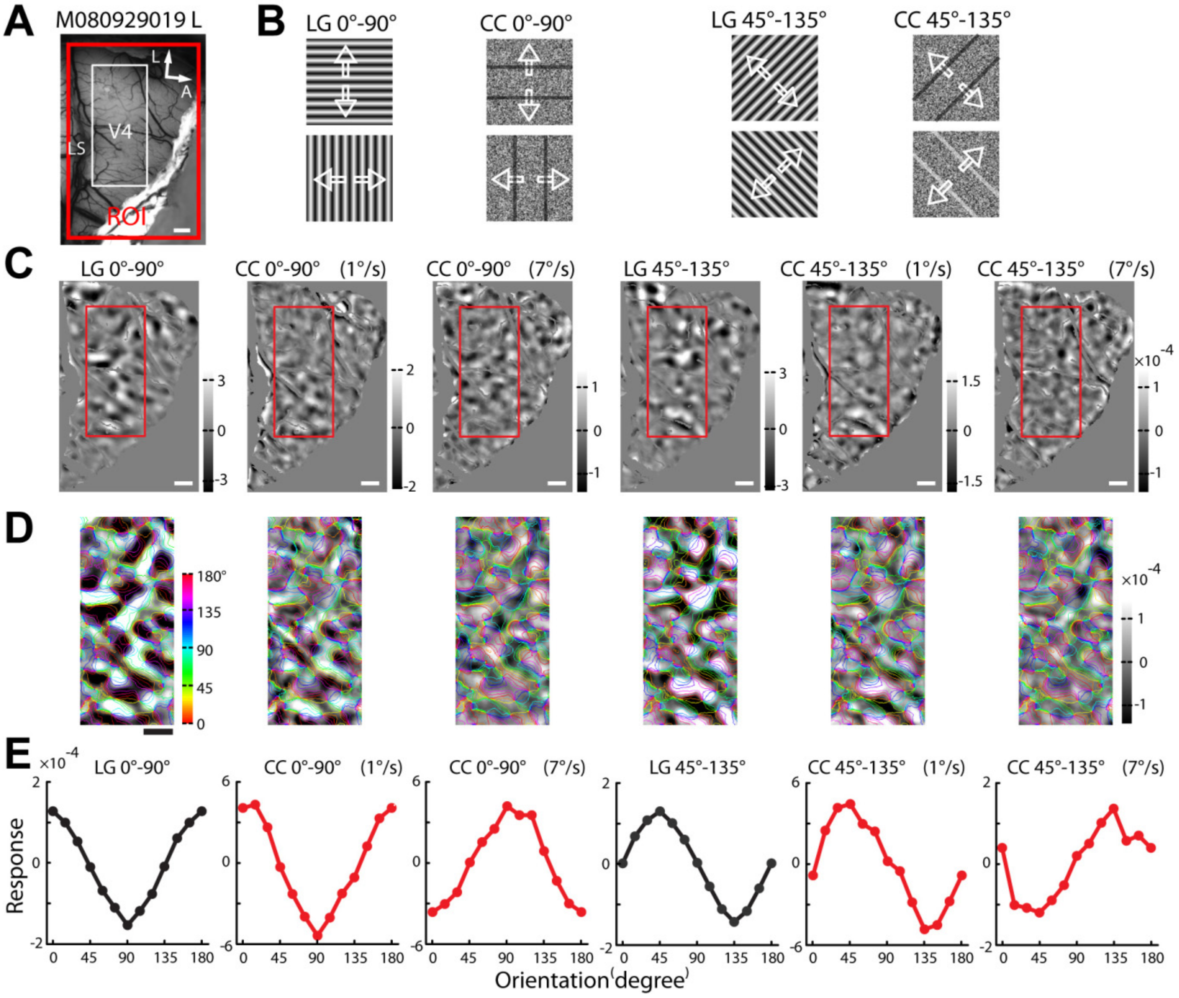

Supplementary Figure 1: Orientation domains in V4 activated by CC stimuli at speeds of 1 and 7 °/s. (**A**) The surface vasculature of V4 ROI in the left hemisphere of macaque 080929019. (**B**) The LG and CC stimulus pairs. (**C**) Differential maps for 0° and 90°, 45° and 135° orientation pairs. (**D**) Differential maps from areas boxed in **C** superimposed with colored iso-orientation outlines derived from the orientation preference map generated with LG stimuli. (**E**) The response profiles depict the orientation preferences of CC stimuli moving at speeds of 1 and 7 °/s.

Supplementary Figure 2

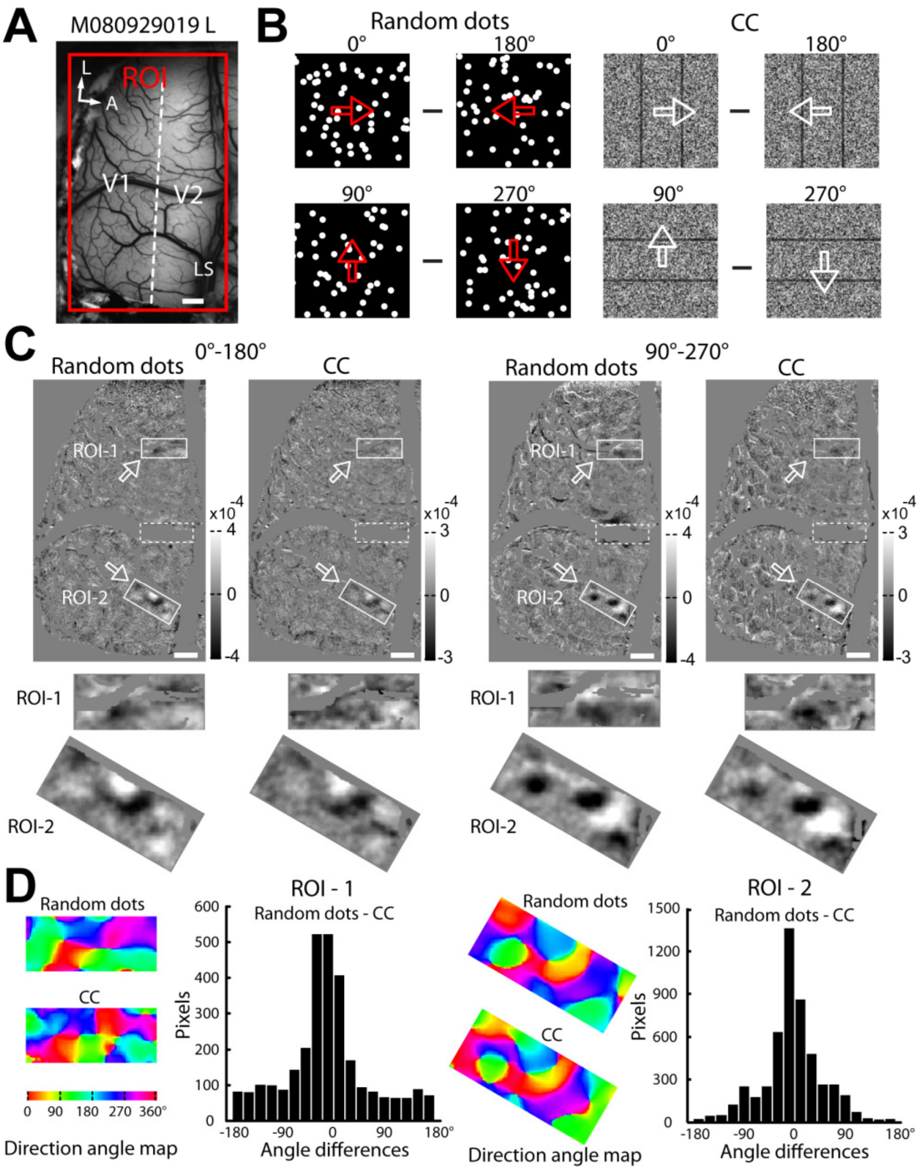

Supplementary Figure 2: The direction-selective domains in V2 activated by CC stimuli at speed 1 °/s. (**A**) The surface vasculature of the ROI of V1 and V2 in the left hemisphere of macaque 080929019. (**B**) The moving random dot and CC stimulus pairs with 0°, 180°, 90°, and 270° directions. (**C**) Differential maps for 0° and 180°, 90° and 270° direction pairs. ROI 1 and 2 illustrate the direction-selective domains activated by pairs with opposite motion directions. No direction domains were activated in V1 by either stimulus. (**D**) The colored direction preference maps. Using the conventional vector-summation method, direction preference maps were generated separately for random dots and for the camouflage-breaking stimuli within the two ROIs. Histograms of the angular differences between the two pairs of direction preference maps peak around 0°.

Supplementary Figure 3

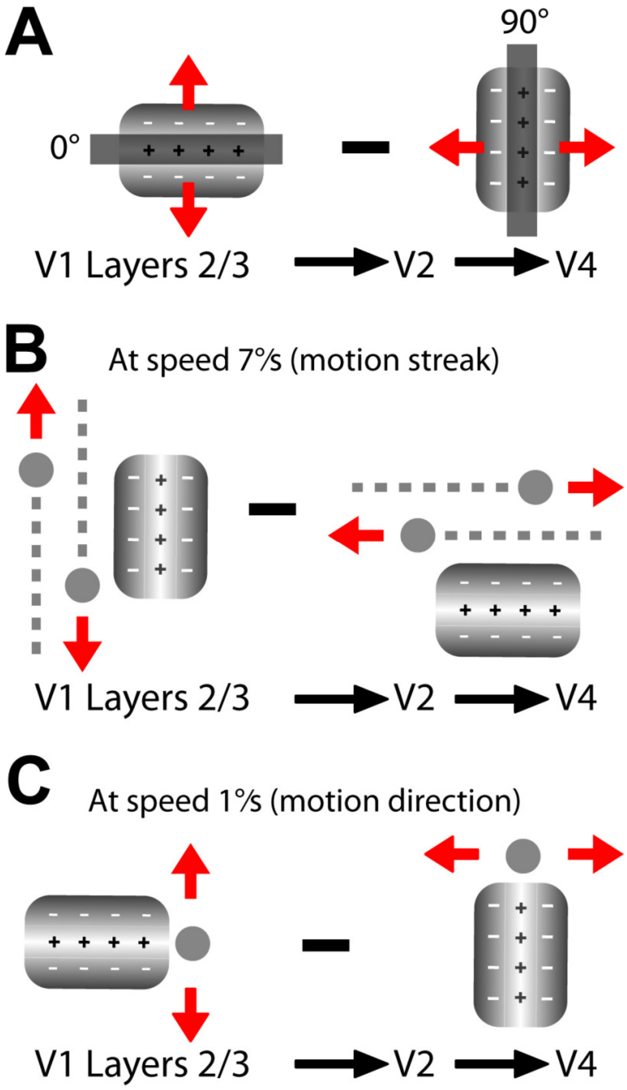

Supplementary Figure 3: Schematic of the presumed populations driving the intrinsic signal responses to camouflage-breaking motion. (**A**) Orientation-selective neurons respond equivalently to both (orthogonal) directions of a grating (LG) / bar stimulus. (**B**) At 7 °/s orientation-selective neurons respond to texture (within CC) or dots stimuli moving parallel to their preferred orientation, i.e. the motion-streak response. (**C**) At 1 °/s orientation-selective neurons respond to both directions of motion orthogonal to their preferred orientation, similarly to the LG stimulus response.

Supplementary Figure 4

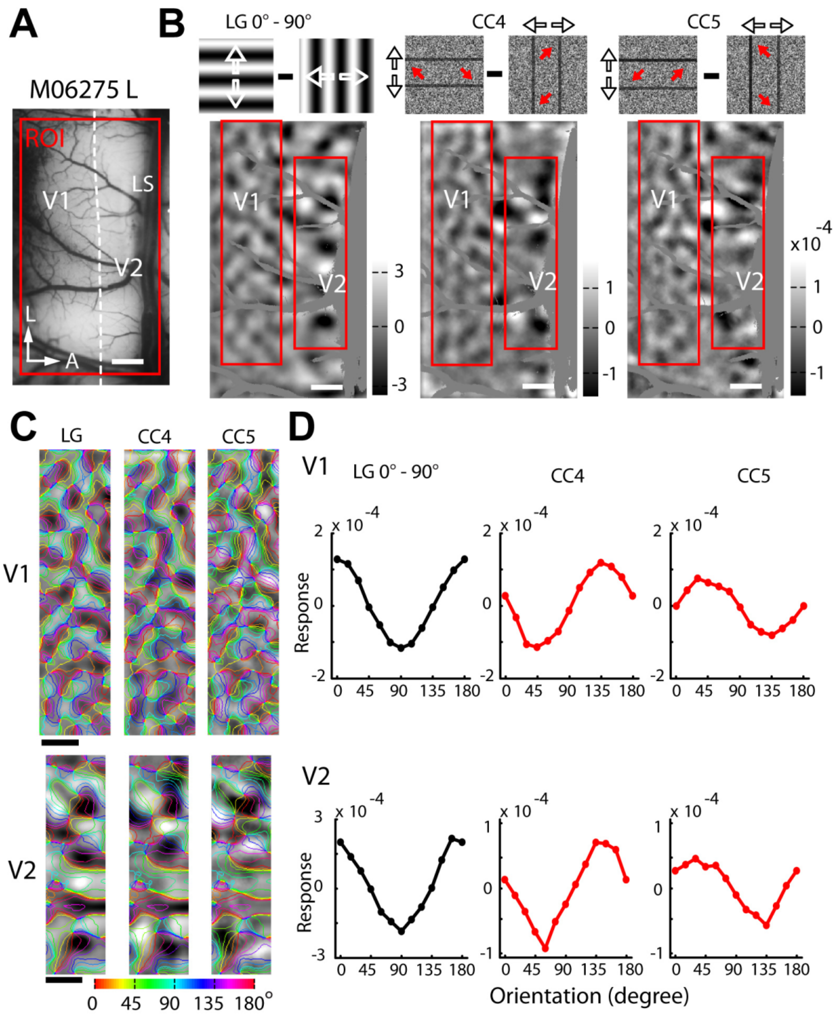

Supplementary Figure 4: Orientation domains activated by camouflage contours with inducer-motion axis 45° off the orientation of the camouflage-breaking contours. (**A**) Picture of the surface vasculature of the left hemisphere of macaque 06275 with ROI of V1 and V2 boxed with red lines. (**B**) Differential orientation maps activated by different stimulus pairs as displayed on top of each map. The arrows outside each panel indicate the moving directions of the global contours while those inside indicate the moving directions of the noise inducers. Note that the motion axes of the moving noise inducers in the CC4 stimuli are perpendicular to those in CC5, but both create kinetic contours with the same orientations. Blood vessels were colored gray. (**C**) Differential maps from V1 (upper panel) and V2 (lower panel) as areas boxed in **B** superimposed with colored iso-orientation outlines derived from the orientation preference map generated with LG stimuli. Pixels covered by blood-vessel masks as shown in **B** were interpolated for clarity. (**D**) Results of response profile analysis. The black and red curves represent LG and CC respectively.

Supplementary Figure 5

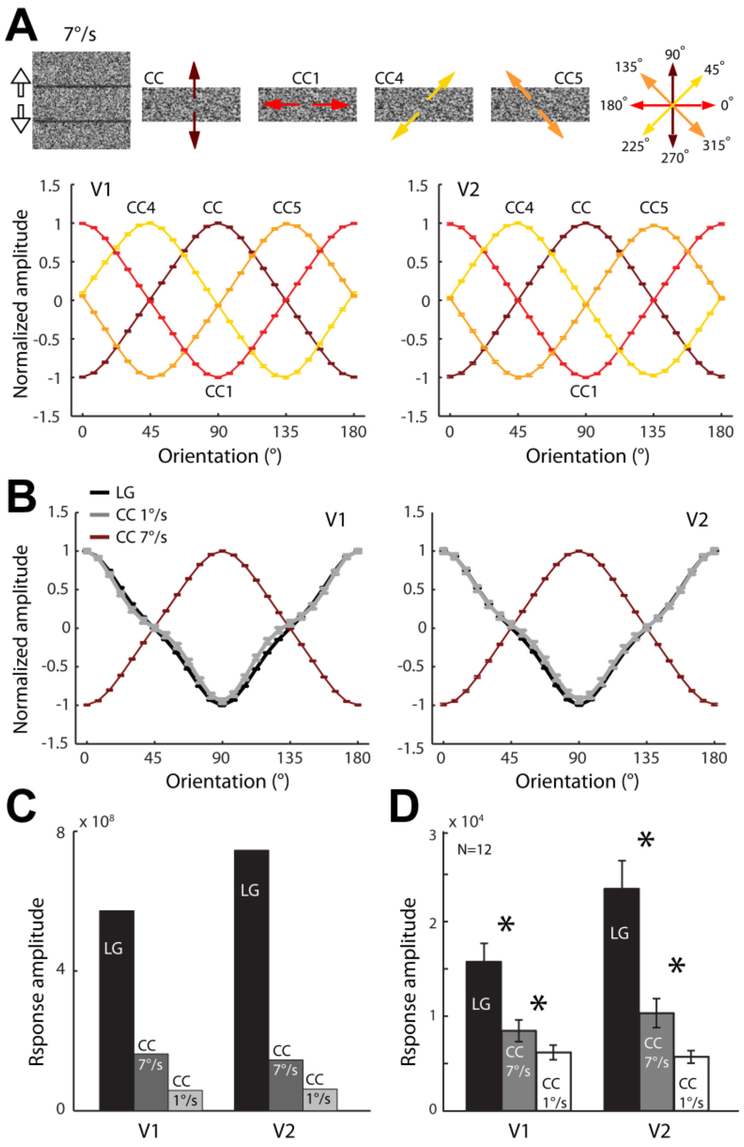

Supplementary Figure 5. Energy model simulation and the response strength for V1 and V2. (**A**) The illustration of CC stimuli (only horizontal orientation displayed) moving in different motion axes. The population response profiles produced by the energy model in response to a pair of CC stimuli with 0° and 90° orientation are presented underneath. Error bars show the S.E.M. over 32 trials. (**B**) The simulated population response profiles by the energy model in response to LG stimuli (0° - 90°) and CC stimuli of the same orientations at speeds 1 and 7 °/s. (**C**) Response magnitudes of V1 and V2 populations produced by the energy model. (**D**) The absolute population response strengths of intrinsic signals (ΔR/R, changes of reflected light) for all stimulus types. This measure was obtained by averaging the maximum responses to each stimulus orientation. N: number of cases. The stars represent significant differences between different stimuli. Error bars represent S.E.M.

Supplementary Figure 6

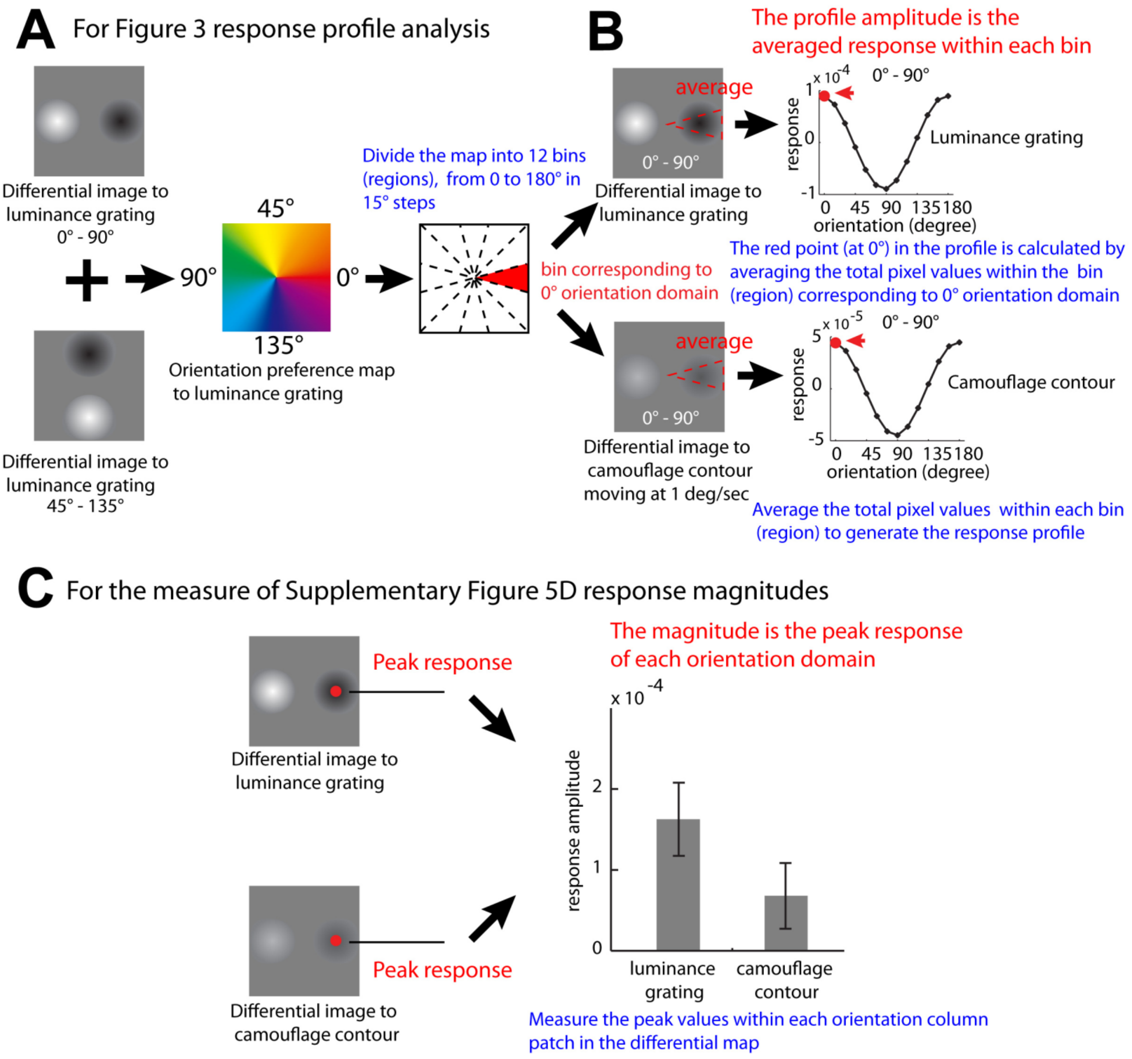

Supplementary Figure 6. Diagram illustrates response profile analysis and the response strength measurement. (**A, B**) The illustration of calculation of average responses within a cortical region to generate orientation response profiles. The cortical region (bin) is defined by luminance gratings. (**C**) The illustration of calculation of peak response within an orientation column patch in the orientation differential map. The peak response is the max of the change of reflected light (Max ΔR/R value).
